# L1 retrotransposition in the soma: a field jumping ahead

**DOI:** 10.1186/s13100-018-0128-1

**Published:** 2018-07-07

**Authors:** Geoffrey J. Faulkner, Victor Billon

**Affiliations:** 1 Mater Research Institute – University of Queensland, TRI Building, Woolloongabba, QLD 4102 Australia; 2 School of Biomedical Sciences, University of Queensland, Brisbane, QLD 4072 Australia; 3 Queensland Brain Institute, University of Queensland, Brisbane, QLD 4072 Australia; 4 Biology Department, École Normale Supérieure Paris-Saclay, 61 Avenue du Président Wilson, 94230 Cachan, France

**Keywords:** LINE-1, L1, Genomics, Retrotransposon, Mosaicism, Neurobiology

## Abstract

Retrotransposons are transposable elements (TEs) capable of “jumping” in germ, embryonic and tumor cells and, as is now clearly established, in the neuronal lineage. Mosaic TE insertions form part of a broader landscape of somatic genome variation and hold significant potential to generate phenotypic diversity, in the brain and elsewhere. At present, the LINE-1 (L1) retrotransposon family appears to be the most active autonomous TE in most mammals, based on experimental data obtained from disease-causing L1 mutations, engineered L1 reporter systems tested in cultured cells and transgenic rodents, and single-cell genomic analyses. However, the biological consequences of almost all somatic L1 insertions identified thus far remain unknown. In this review, we briefly summarize the current state-of-the-art in the field, including estimates of L1 retrotransposition rate in neurons. We bring forward the hypothesis that an extensive subset of retrotransposition-competent L1s may be de-repressed and mobile in the soma but largely inactive in the germline. We discuss recent reports of non-canonical L1-associated sequence variants in the brain and propose that the elevated L1 DNA content reported in several neurological disorders may predominantly comprise accumulated, unintegrated L1 nucleic acids, rather than somatic L1 insertions. Finally, we consider the main objectives and obstacles going forward in elucidating the biological impact of somatic retrotransposition.

## Background

Transposable elements (TEs) and their mobilization in somatic cells were first described by Barbara McClintock’s celebrated research on *Ac*/*Ds* loci in maize [1]. In the intervening 70 years, somatic transposition (“cut-and-paste”) and retrotransposition (“copy-and-paste”) of TEs has been reported throughout the tree of life, including, for example, in plants [2, 3], insects [4–7], rodents [8–10] and primates [11]. By definition, mosaic TE insertions are present in at least one, but not all, cells from an individual. New TE insertions, or the deletion of existing TE insertions [12], may generate germline as well as somatic mosaicism. Indeed, the primary milieu for heritable LINE-1 (L1) retrotransposition in mammals is the early embryo [13], where new L1 insertions can enter the germline and contribute genetic diversity to offspring [14–17] whilst potentially also causing somatic mosaicism in the original host [8, 10, 11, 18]. As embryonic development continues, L1 mobilization appears to become more lineage-restricted, perhaps to the extent that only neurons and their progenitor cells support endogenous L1 activity [19–21]. Somatic L1 retrotransposition may therefore be an evolutionary byproduct of TEs being active in the developmental niches most likely to spread new copies of themselves to as many germ cells as possible, combined with an inability to prohibit L1 activity in some committed lineages [20–22]. We presently lack compelling evidence to reject the null hypothesis that somatic retrotransposition in normal cells is of little consequence to human biology. Intriguing experimental data do however show that L1 activity is elevated coincident with environmental stimuli [23–25] and, more extensively, in psychiatric and neurodevelopmental disorders [26–29]. As a summary view, we propose that retrotransposons can cause somatic mosaicism in mammals, yet the frequency, spatiotemporal extent, biological impact, and molecular processes regulating this phenomenon remain poorly defined.

## L1 retrotransposons

**Fig. 1:**
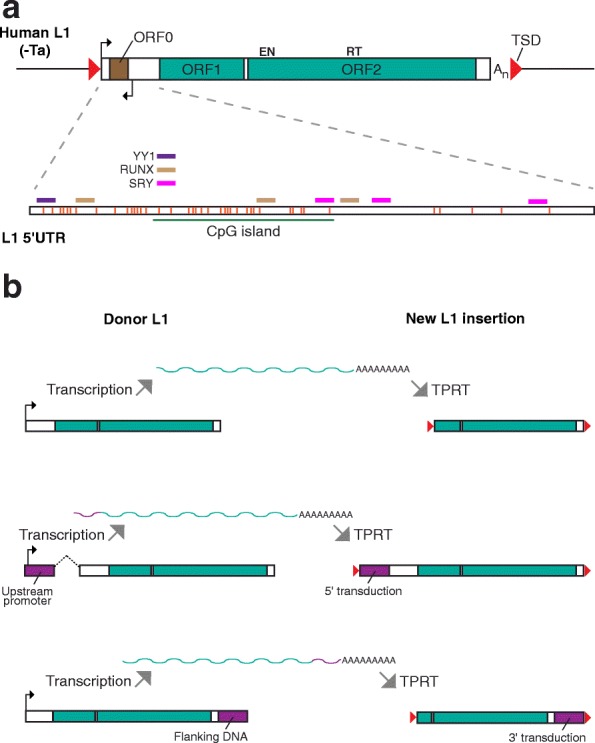
L1 retrotransposon structure and mobilization scenarios. **a.** A human L1-Ta element (top) is 6 kb in length and encodes two protein-coding open reading frames (ORF1 and ORF2) flanked by 5′ and 3′ UTRs. New L1 insertions are typically flanked by a 3′ polyadenine (A_n_) tract as mRNA polyadenylation is critical to efficient L1 retrotransposition [61, 62]. An antisense open reading frame (ORF0, brown rectangle) is located in the 5′UTR and may facilitate retrotransposition [209]. ORF2p possesses endonuclease (EN) and reverse transcriptase (RT) activities [44, 45]. The L1 is transcribed from 5′ sense (canonical) [47] and antisense [208] promoters, as indicated by black arrows. Target-primed reverse transcription (TPRT) typically generates short target site duplications (TSDs, indicated by red triangles) flanking new L1 insertions [44, 46, 64, 66]. A closer view of the L1 5′UTR (bottom) indicates YY1 (purple rectangle), RUNX (brown rectangle) and SRY family (e.g. SOX2, pink rectangle) transcription factor binding sites [22, 69, 207]. Numerous CpG dinucleotides (orange bars) occur throughout this region and, at a point of sufficient density, form a CpG island (green line) that is regulated by a complex including MeCP2, HDAC1 and HDAC2 [27, 47, 75, 105]. **b.** Example L1 mobilization scenarios. Top: A donor L1 is transcribed from its canonical promoter, generates a polyadenylated mRNA, and is retrotransposed via TPRT, generating a new L1 insertion that is 5′ truncated. Middle: Transcription initiated by a promoter upstream of the donor L1 reads through into the L1 and generates a spliced (dotted line) mRNA. As a result, the new L1 insertion carries a 5′ transduction. Bottom: Transcription initiates as directed by the canonical promoter but reads through the L1 polyA signal to an alternative downstream signal. Reverse transcription and integration of this mRNA generates a 5′ truncated L1 insertion flanked by a 3′ transduction. Note: the monomeric promoters of the active mouse L1 subfamilies (T_F_, G_F_, A) are very different in their structure, and potentially their regulation, than the human L1-Ta promoter. Aspects of the figure are adapted from previous works [35, 290]

Several retrotransposon families are currently mobile in mouse and human [16, 30–34]. In this review, we focus on L1 as the only element proven, by multiple orthogonal approaches, to retrotranspose in somatic cells in vivo [35]. Annotated L1 sequences occupy nearly 20% of the human and mouse reference genomes [36, 37]. Although more than 500,000 L1 copies are found in either species, only ~ 100 and ~ 3000 retrotransposition-competent L1s are found per individual human [38, 39] or mouse [40–43], respectively. A full-length, retrotransposition-competent (donor) L1 is 6-7kbp in length, contains two open reading frames encoding proteins strictly required for retrotransposition (ORF1p and ORF2p) and is transcriptionally regulated by an internal 5′ promoter [44–47] (Fig. 1). Retrotransposition requires transcription of a polyadenylated mRNA initiated by the canonical L1 promoter, followed by export of the L1 mRNA to the cytoplasm and translation, yielding ORF1p and ORF2p [48–50]. Due to *cis* preference, the L1 mRNA is bound by ORF1p and ORF2p to form a ribonucleoprotein (RNP) that can re-enter the nucleus [51–60]. Reverse transcription of the L1 mRNA by ORF2p, primed from a genomic free 3′-OH generated by ORF2p endonuclease activity [44, 45, 58, 61–63], followed by removal of the L1 mRNA from the intermediate DNA:RNA hybrid, and second strand DNA synthesis, generates a new L1 insertion. This molecular process, termed target-primed reverse transcription (TPRT), was first established by a seminal study of *Bombyx mori* R2 retrotransposons [64]. If generated via TPRT, new L1 insertions usually carry specific sequence features, including short target site duplications (TSDs) and a polyadenine (polyA) tail (Fig. 1), and integrate into the genome at a degenerate L1 endonuclease motif [44, 46, 65–67]. These TPRT hallmarks can be used to validate somatic L1 insertions [67]. A fraction of new L1 insertions transduce DNA from the genomic flanks of their donor L1 to the integration site, facilitating identification of the donor sequence (Fig. 1) [36, 60, 68–72]. 5′ truncation, internal mutations and the acquisition of repressive epigenetic marks can reduce or abolish the retrotransposition competence of new L1 insertions [47, 69, 73–77]. Finally, L1 can mobilize other cellular RNAs in *trans*, including those produced by *Alu* and SVA retrotransposons, adding to L1-driven genome sequence variation [31, 32, 34, 78, 79].

The vast majority of highly active, or “hot”, human donor L1s belong to the L1-Ta subfamily [33, 38, 39, 80–83] and fewer than 10 hot L1s are present in each individual [39]. These hot elements are usually highly polymorphic, with millions of donor L1 alleles potentially yet to be found in the global population [14, 38, 39, 76, 83–85]. Approximately 1 in 150 individuals harbors a new L1 insertion [86]. By contrast, three L1 subfamilies (T_F_, G_F_, A), defined by their monomeric 5′ promoter and ORF1 sequences, remain retrotransposition-competent in the mouse germline [16, 17, 40–43, 87–90]. At least 1 in 8 pups carries a new L1 insertion in inbred C57BL/6 J mice [13, 18]. As for human L1s, internal mutations can strongly influence the mobility of individual mouse L1s [40, 72, 91, 92]. Although the mouse genome contains many more full-length L1s with intact ORFs than the human genome [93], it is unknown whether mouse L1 retrotransposition potential is concentrated in a similarly small proportion (< 10%) of elements. The distinct promoter sequences driving L1 transcription in mouse and human, and associated differences in their regulation, may also result in divergent spatiotemporal patterns of L1 expression.

Many, if not most, new L1 insertions are unlikely to generate a phenotype [94]. L1-mediated mutagenesis can nonetheless severely impact the functional products of genes [95] and, presumably as a result, host cells have multiple layers of regulation that limit L1 retrotransposition (Fig. 1, Table 1), including via epigenetic control of the L1 promoter [20, 27, 96–108] (for relevant recent reviews on L1 host factors and L1 mutations in disease, please see [109–115]). Even so, L1 mRNA expression and retrotransposition can occur in the pluripotent cells of the early mouse and human embryo, enabling somatic and germline L1 mosaicism prior to lineage commitment [8, 10, 11, 18, 104, 116–121].

**Table 1:** Host factors that regulate L1 mobilization

Several proteins inhibit L1 transcription. **MeCP2** binds methylated cytosines in the CpG island core of the L1 promoter [27, 47, 75, 105]. MeCP2 occupancy prevents cytosine hydroxymethylation and L1 de-repression by the activator **TET1**, and facilitates the recruitment of methyltransferases affixing the repressive chromatin mark H3K9me3 [104, 285, 286]. Other factors such as **KAP1**, the **HUSH** complex and **MORC2** bind and silence full-length L1s, including those located in euchromatic genomic regions, again via deposition of repressive marks [96, 103, 106, 285]. Another key repressor, **SOX2**, is a transcription factor that inhibits neuronal gene and L1 expression during development. Neuronal maturation requires SOX2 down-regulation, which may explain the potential specificity of L1 mobilization in neurons [20, 22]. By contrast, the transcription factors **RUNX3** and **YY1** assist L1 transcription and retrotransposition [69, 207]. Although the mechanism for L1 activation by RUNX3 is unclear, YY1 appears to direct transcriptional initiation to the correct (+ 1) L1 start site, and may also support loops involving the L1 promoter and enhancer elements [69, 287]. Numerous factors [112] also repress L1 at the post-transcriptional level, and may each do so in multiple ways. For example, the adenosine deaminase **ADAR1** inhibits L1 mobilization via editing dependent and independent activities, which could involve binding to the L1 RNP [57, 245, 288, 289]. The exonuclease **TREX1** has been shown to inhibit retrotransposition in vitro by depleting L1 ORF1p and altering its subcellular localization [247], whereas **SAMHD1** inhibits L1 mobilization by limiting the availability of intracellular nucleotides and other mechanisms [244, 246]. Finally, during L1 integration, TPRT intermediate DNA-RNA hybrids are targeted by host factors, such as **APOBEC3A**, which deaminates transiently exposed single-stranded DNA [139].	

## Engineered L1 mobilization during neuronal differentiation

**Fig. 2:**
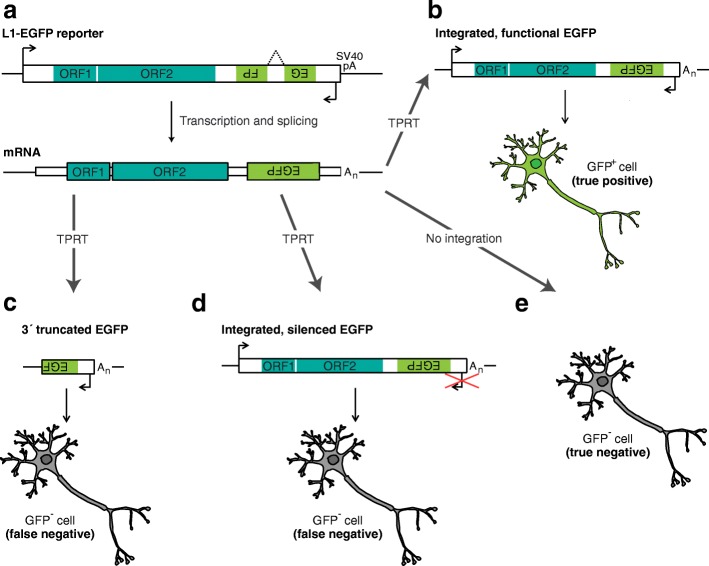
Interpreting results from the engineered L1-EGFP reporter assay. **a.** The L1-EGFP reporter gene [123] comprises a full-length human or mouse L1 (e.g. [41, 122, 291]) tagged with a cassette incorporating EGFP and its promoter in the opposite orientation to the L1, followed by an SV40 polyA signal. Transcription of the combined L1-EGFP reporter, followed by splicing (dotted line) of an intron in the EGFP gene, prepares the L1-EGFP mRNA for reverse transcription and integration into the genome via target-primed reverse transcription (TPRT). The L1-EGFP reporter has been introduced in vitro as a plasmid [19–21, 126, 171] and also as a rodent transgene [8, 9, 21, 27, 116]. **b.** Successful TPRT-mediated retrotransposition of the engineered L1 mRNA yields an intact EGFP gene, leading to GFP^+^ cells (true positives). **c.** Mobilization of the engineered L1 mRNA may occur through TPRT but, due to severe 5′ truncation removing the L1 entirely, or 5′ inversion/deletion [95, 292] the EGFP gene may be incompetent at its 3′ end, and therefore retrotransposition results in GFP^−^ cells (false negatives). **d.** The engineered L1 mRNA may be retrotransposed, yielding a functional EGFP gene, but the EGFP promoter is epigenetically silenced [126], leading to GFP^−^ cells (false negatives). PCR-based assays targeting the EGFP splice junction can, however, identify instances where successful retrotransposition is not marked by EGFP expression [19, 46, 123, 126]. **e.** Finally, retrotransposition of the engineered L1 mRNA may simply have not occurred in GFP^−^ cells (true negatives)

Neurons and their precursor cells present an exception to L1 restriction in normal committed lineages [19]. The first experimental evidence of L1 retrotransposition in the neuronal lineage was obtained from an engineered system where a human L1 (L1_RP_ [122]) tagged with an EGFP reporter gene [116, 123] was introduced into cultivated rat neural cells, and into mice as a transgene (Fig. 2) [21]. Strikingly, GFP^+^ neurons were found in transgenic mice whilst few, if any, GFP^+^ cells were found in other somatic cell types [21]. Using a different human L1 (L1.3 [124, 125]) tagged with a similar EGFP cassette, our laboratory has recently recapitulated this result (Bodea et al., *unpublished data*). The L1-EGFP reporter system has been shown to readily mobilize in embryonic stem cells, neural stem cells, neuronal precursor cells, and post-mitotic neurons [19–21, 119, 121], indicating potential for endogenous L1 activity at various points of neuronal differentiation in vivo.

Engineered L1-EGFP insertions lacking an intact EGFP sequence due to severe 5′ truncation, as well as those affected by epigenetic silencing of the heterologous promoter driving EGFP expression [19, 21, 126], can result in GFP^−^ cells where retrotransposition has actually taken place (Fig. 2) [126]. As a further caveat, an EGFP-tagged human L1 introduced as a transgene is also likely not subject to the same host factor control as exerted in its native genome. Engineered L1 reporter systems [9, 10, 46, 71, 90, 127, 128] can still provide proof-of-principle evidence that the L1 machinery may enact retrotransposition of L1 and other TEs [31, 32, 34, 79, 129] in a given spatiotemporal context, although, to our knowledge, *Alu* or SVA *trans* mobilization by L1 is yet to be demonstrated in primary neurons or neuronal precursor cells. Engineered L1 systems have nonetheless predicted, with substantial success, L1 activity in cells where endogenous L1 mobilization was later confirmed by genomic assays, as for example in the case of the brain.

## What is the frequency of endogenous L1 retrotransposition in neurons?

Endogenous L1 retrotransposition is established to occur in mammalian neurons (for reviews, see [35, 67, 130–132]). This conclusion is based on genomic analysis of “bulk” brain tissue [20, 133] and individual neural cells, with the latter requiring whole-genome amplification (WGA) [134–137] or reprogramming via nuclear transfer followed by clonal cell amplification [138]. Exemplary somatic L1 insertions reported to date include two events carrying 5′ or 3′ transductions [36, 68], which were recovered from individual human cortical neurons through WGA followed by whole-genome sequencing (WGS) [136]. Subsequent insertion site-specific PCR amplification and capillary sequencing revealed structural hallmarks consistent with retrotransposition by TPRT [136]. Analyses employing WGA and targeting human L1-genome junctions have also recovered neuronal L1 insertions [134, 135, 137]. Using an orthogonal approach, and in mouse, Hazen et al. applied WGS to stem cell clones reprogrammed via nuclear transfer of olfactory neuron nuclei, and again found somatic L1 insertions mediated by canonical TPRT [138]. Impressively, this work identified 4 somatic L1 insertions in only 6 reprogrammed neuronal clones, with a false negative rate of at least 50% [138] as mouse L1 3′ ends are depleted in Illumina sequencing [18, 35]. These and other genomic analyses of neuronal genomes have thus far yielded results highly congruent with experiments employing the L1-EGFP reporter in vitro and in transgenic animals [19–21]. Together with somatic L1 insertions that may accumulate earlier in development [11, 18, 136], these data suggest that L1 mosaicism occurs relatively often in the mammalian brain. The expected frequency of L1 retrotransposition in neurons is however debated [35, 132, 134, 137] and depends on multiple factors, such as the methods used for WGA, library preparation and sequencing, how false positive and false negative rates are calculated, how insertions are validated, as well as the species, brain region and neuronal subtype being analyzed. Importantly, L1 insertion mapping strategies only find *completed* retrotransposition events. Host factors may eliminate TPRT intermediates in neurons before integration is fully executed (Table 1) [29, 139, 140] and, for this reason, the frequency of attempted somatic L1 retrotransposition events may be higher than what is found by studies of either endogenous or engineered L1 mobilization.

Current estimates of the L1 retrotransposition rate in human neuronal cells range from 0.04 to 13.7 L1 insertions per neuron [35]. In this context, what is a “low” or “high” frequency? If we assume that the typical human brain contains ~ 90 billion neurons [141], and apply a conservative denominator of the current lowest estimate of 0.04 unique events per neuron, we would still expect at least 3.6 billion somatic L1 insertions per human brain, and many more events may be shared by multiple cells. Should this be considered as a low rate? Firstly, brain cells are far more physiologically and functionally interdependent than myocytes, hepatocytes, fibroblasts and other somatic cell types found in the body. Highly interconnected neuronal networks may hence be disproportionately impacted by mutations in “node” cells [142, 143]. Secondly, rather than occurring randomly throughout the genome, somatic L1 insertions may be found at a significantly higher rate in neuronally expressed genes [21, 133, 137], although at this stage the separation of potential endogenous L1 insertional preference from post-insertion selection and detection bias is challenging. Thirdly, neurodevelopmental disorders may be caused by somatic mutations penetrating less than 10% of neurons from a given brain region [144–146] and, moreover, of the two neuronal L1 insertions to undergo lineage tracing thus far, one was found in up to 1.7% of neurons sampled from the cortex [136]. Fourthly, L1 insertions are only one of several types of genomic variant encountered in the brain [147]. These include aneuploidy and other forms of copy number variation (CNV) [148–150], as well as single nucleotide variants (SNVs) [151, 152]. Analyses of bulk genomic DNA extracted from brain tissue have elucidated somatic *Alu* and SVA insertions [133, 153], while a single-cell WGS analysis of a relatively small set of cortical neurons did not find somatic variants attributed to either *trans* mobilized retrotransposon family [136]. L1 insertions are far larger than an SNV and perhaps carry an average effect size more similar to that of a copy number or structural variant, depending on the genomic and biological context where the variant occurs. These considerations suggest that, with the improving resolution and expanding scale of single-cell genomic analysis applied to brain tissue, somatic L1 insertions causing a neuronal or cognitive phenotype will be identified in the coming years. At present, however, very few neurons, almost exclusively from a handful of neurotypical individuals, have been interrogated for endogenous L1 retrotransposition events. Single-cell genomic experiments that exhaustively survey neuronal subtypes, from numerous individuals and brain regions, are required to define the typical range of neuronal L1 retrotransposition frequency in humans [147]. By also elucidating the genomic locations of new L1 insertions, and their functional effects, these future studies should greatly inform our view of whether L1-driven mosaicism has the potential to be a phenomenon of biological importance, building on foundational evidence now showing that endogenous L1s can jump in the brain.

## L1 retrotransposition in non-neuronal brain cells

Somatic L1 insertions have been found in hippocampal glia by recent single-cell genomic analyses [134, 137]. By contrast, experiments based on cultured glial cells and the L1-EGFP system have suggested that retrotransposition in glia is uncommon [21]. One possible explanation for the presence of somatic L1 insertions in glia is that neural stem cells can accommodate retrotransposition events prior to neuronal commitment, leading to occasional L1 insertions in multipotent precursor cells that ultimately commit to the glial lineage [20]. Unlike most neuronal populations, glia can also divide and regenerate in response to injury [154, 155] and this capacity for cell cycling may facilitate retrotransposition [59, 156–158]. Comparisons of L1 retrotransposition rate in glia versus neurons are, for these reasons, not straightforward. Even if, on average, they accumulate fewer L1 insertions than neurons [137], individual glia can oversee more than 100,000 synapses [159] and impact the functional output of the neurons they support [160]. To speculate, one can therefore envisage a situation where a somatic L1 insertion in a glial cell that supports or protects a large number of neurons could, by extension, alter the functional properties of at least some of those neurons, potentially adding to any direct impact of neuronal L1 insertions [131]. This may be disproportionately likely in pathologic conditions, such as autoimmune diseases where L1 expression in astrocytes for example may be unusually high [29]. It should again be noted, however, that a molecular or biological phenotype is yet to be demonstrated for any somatic L1 insertion arising in a neural cell. Moreover, glial proliferation and regeneration may buffer cells from the potential consequences of somatic L1 insertions, lessening the likelihood of downstream changes to neuronal circuits. Further experimental evidence is required to conclusively demonstrate that somatic L1 insertions can arise in committed glia, as opposed to multipotent progenitor cells. Similarly, L1 retrotransposition is heavily influenced by cellular host factors (Table 1), but we know little about the host factors that regulate L1 in neurons, as compared to those active in glial cells. Thus, it is likely that the L1 mobilization rate in glia and neurons, including neuronal subtypes, may be reliant upon the differential expression of L1 regulatory proteins in these cells.

## Somatic retrotransposition outside of the brain?

To our knowledge, no single-cell genomic analysis of somatic retrotransposition has been reported for mammalian organs other than the brain, although a few immortalized skin cells have been surveyed by WGS without a specific search for mosaic TE insertions [151]. This presents a major gap in the field as, at present, we cannot ascertain whether endogenous L1 retrotransposition really is enriched in the brain or occurs, for instance, in liver, heart or skin at a rate resembling that observed for neurons. Bulk sequencing approaches have found isolated examples of likely somatic L1 insertions in normal liver [161] and gastrointestinal tract [162–165] tissues of cancer patients, as well as mosaic L1 insertions found in various adult mouse tissues but arising prior to gastrulation [18]. By contrast, a bulk WGS analysis of 10 clonal cell populations expanded from single skin fibroblasts identified no somatic L1 insertions that could be traced to a parental cell [166]. Transgenic L1-EGFP animals also present very few GFP^+^ cells outside of the brain and gonads [9, 21] and, when employed in vitro, the L1-EGFP reporter retrotransposes consistently in neural progenitor cells and post-mitotic neurons [19–21] but not mesenchymal or hematopoietic stem cells [19].

Taken together, these observations support a model where L1 insertions arising in the early embryo may generate low complexity mosaicism in multiple organs, complemented by ongoing retrotransposition in brain cells. Other adult cell types may also support somatic retrotransposition. However, single-cell genomic analyses of post-mortem, non-brain tissues from human individuals not affected by cancer or other relevant diseases will be required in the future to definitely assess endogenous L1 retrotransposition outside of the brain. That L1 mobilizes frequently in many epithelial tumors [72, 161, 162, 164, 165, 167–174], but rarely in brain tumors [168, 169, 175, 176], suggests that dysplastic epithelial cells may specifically support L1 activity. The discovery of somatic L1 insertions in the pathologically normal cells of organs where tumorigenesis has occurred reinforces this conclusion [161–165] but falls short of demonstrating retrotransposition in a healthy organ. Nonetheless, cancer has provided the only examples thus far of somatic retrotransposition causing a clinical or molecular phenotype [161, 163, 167, 170, 171], and has greatly informed our understanding of L1 regulation in vivo (for relevant reviews, please see [109, 114, 177]).

## Transposition in the fly brain

L1 and L1-like retrotransposons are found throughout the eukaryotic tree of life [93]. In animals, somatic TE insertions have been almost exclusively reported in human and rodent tissues and experimental systems [35]. The main exception is *Drosophila*, where R2, a highly site-specific, L1-like retrotransposon, and *gypsy*, an endogenous retrovirus found to often integrate into specific genomic hotspots, have been found to mobilize in somatic cells, including neurons [4–7, 178–180] (for a review, see [181]). Targeted PCR and resequencing, and orthogonal reporter assays, have each indicated retrotransposon integration (e.g. R2 into rRNA genes [64, 182], *gypsy* into the *ovo* gene [183, 184]). However, in contrast to mammalian systems, genome-wide attempts to map endogenous TE mobilization in fly somatic cells have to date not corroborated the aforementioned data obtained from reporter assays. For example, Perrat et al. applied a shallow WGS analysis to pooled fly embryos, brain tissue, and pooled olfactory (αβ) neurons purified from mushroom body, generating an estimate of 129 somatic TE insertions per αβ neuron [185]. However, a subsequent and thoughtful WGS analysis of additional αβ neurons, using improved sequencing depth but still incorporating pooled neuronal material, and analyzing the evolutionary age of mobilized TEs, found no evidence for somatic TE transposition in the fly brain [186]. This second study reversed the earlier conclusion of widespread transposon-mediated genomic heterogeneity in the fly brain [185] and leaves the question of somatic transposition rate in fly unresolved. Interestingly, through additional analyses, the authors also challenged previous findings of increased transposition rate in ageing neurons [5] and ovaries obtained from dysgenic hybrids [187] but did not reanalyze the Perrat et al. sequencing data [186]. Given the aforementioned R2 and *gypsy* experiments [4–7], we would postulate that a single-cell genomic analysis of fly neurons, with appropriate genotypic controls (i.e. non-brain tissue from the same fly) would identify somatic transposition events. These would likely occur at a lower frequency than first reported by Perrat et al. but, given the extensive array of mobile TE families in the *Drosophila* genome [188], perhaps at a higher frequency than seen in mammalian neurons thus far, and with the caveat that somatic transposition in different fly strains may vary greatly in incidence [189]. Aside from the available data obtained from some mammals and insects, it is currently unknown whether TEs can mobilize in the brain (or other somatic tissues) of other animals. The future discovery of somatic retrotransposition in additional species may greatly assist in elucidating any functional consequences of TE-derived mosaicism in neurons.

## Donor L1s active in somatic cells: Different LINEs to retrotransposition

**Fig. 3:**
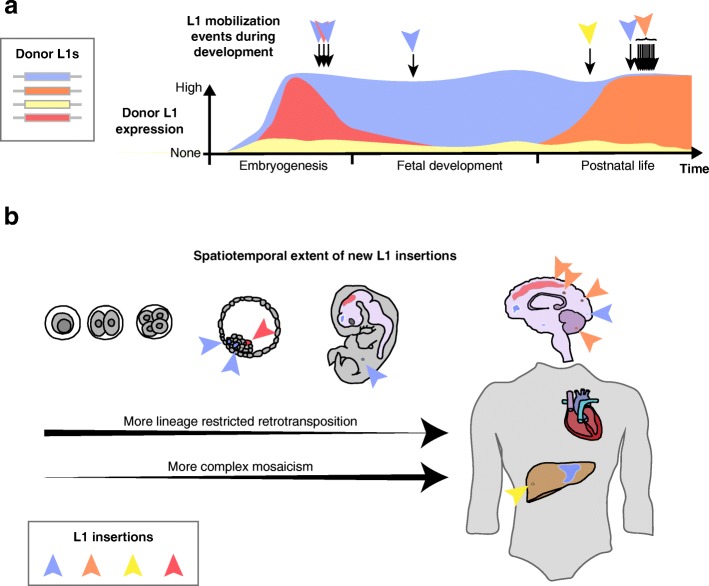
Somatic retrotransposition can cause complex genomic mosaicism. **a.** Donor L1 expression and mobilization during development. A handful of L1 copies from each individual are highly active, or hot, when tested in vitro [38, 39]. Four scenarios for donor L1s mobilizing in vivo are illustrated here. Most L1s are repressed [105] during development and do not mobilize, except perhaps due to exceptional circumstances, such as the availability of an active upstream promoter (e.g. yellow donor L1) [36]. L1 promoter de-repression can however occur during development, either transiently (e.g. red and orange donor L1s) or durably (e.g. blue donor L1), leading to L1 mRNA and RNP accumulation. Retrotransposition enacted by the L1 machinery occurs as a function of donor L1 activity in a given spatiotemporal context (blue, red, orange and yellow arrowheads, matching each donor L1). **b.** The developmental timing of a given retrotransposition event impacts how many mature cells carry the new L1 insertion. Early embryonic L1 mobilization events (e.g. blue and red cells indicated by arrowheads and matching donor L1s by color) may be carried by numerous descendent cells, possibly in different tissues [18]. By contrast, L1 insertions arising later in development (indicated by orange, blue and yellow arrows) are more restricted in their spatiotemporal extent, and may be found in just one cell (e.g. a post-mitotic neurons). The resulting somatic genome mosaicism may disproportionately impact the brain [19–21, 23, 25, 27, 133–138], although further work is required to test whether other organs, such as the liver, also routinely carry somatic L1 insertions [72, 161]

As a rule, L1 epigenetic repression is thought to be established during early gastrulation and maintained thereafter to block L1 mobilization (Fig. 3) [19, 20, 117, 119, 190]. DNA methylation of a CpG island [191] present in the human L1 5′UTR (Fig. 1) is particularly associated with inhibition of L1 expression [98, 103, 192, 193], at least based on relationships between the methylation and transcriptional output of L1 subfamilies, such as L1-Ta [19, 20, 118, 121]. The expression of mouse L1 subfamilies is also inversely correlated with their DNA methylation level [99, 104, 194]. Despite being methylated, full-length L1s are expressed, at varying abundances, in mature somatic tissues [163, 195, 196]. One explanation for this discrepancy is that individual L1s may be regulated in a manner distinct to that of their corresponding L1 subfamily [72, 84]. For example, while genome-wide L1-Ta subfamily mRNA expression may be low in a given context, an individual L1-Ta copy could be highly expressed due to the local demethylation of its promoter. It follows that some donor L1s appear to mobilize in embryonic cells contributing to the germline and in somatic cells at very different efficiencies [85] and present highly variable levels of transcription and mobilization in various cancer cells [84, 174]. Adding to this heterogeneity, individual donor L1s may have multiple alleles that mobilize at disparate rates [76, 83], can be heterozygous or homozygous at a given genomic locus, potentially impacting their regulation, and be fixed or polymorphic in the global population. Repressive epigenetic marks are also not the only means by which L1s are silenced by the host genome (Table 1) [112]. General rules for the genome-wide regulation of an L1 subfamily likely do not apply equally to all L1s in that family and therefore any mechanistic explanation for somatic L1 retrotransposition may rely on locus-specific resolution of L1 repression or activation [72, 84, 163, 171]. As a result, L1 expression and retrotransposition in the germline and in somatic cells are likely to vary considerably between individuals.

Provided these caveats and considerations, we would propose multiple proven or hypothetical scenarios for L1 to escape epigenetic repression and contribute to somatic genome mosaicism. Firstly, many donor L1s are indeed likely to be active in the early embryo (Fig. 3, red scenario) and then repressed in somatic cells, based on DNA methylation patterns observed for the human L1-Ta family overall [19, 20, 118, 121] and, consistently, for several individual hot L1s [121]. Embryonic L1 insertions arising from these elements can be carried through development to generate somatic mosaicism [11, 18]. Secondly, a given donor L1 may be expressed in the embryo and never fully repressed in mature tissues (Fig. 3, blue scenario). One potential example of this was provided by an L1 on Chromosome 17 [38] that was demethylated and expressed in a colorectal tumor, and also the matched normal colon [163]. This donor L1 is a relatively new polymorphism (minor allele frequency 0.0036), is hot for retrotransposition in vitro [38] and is therefore likely to still be mobile during embryogenesis or in the committed primordial germline [18]. Thirdly, a donor L1 may be repressed in the embryo but is found in a genomic locus that does not undergo methylation in differentiated tissues (Fig. 3, orange scenario). A likely example of this is an L1 found on Chromosome 22 that is very active in epithelial tumors [72, 171, 174, 197, 198] but almost inactive in the human germline and in cultured cells [39, 85]. Interestingly, this element is intronic to the gene *TTC28*, which is highly transcribed in epithelial cells and organs where neoplasia often supports retrotransposition of the donor L1 [174, 199] alongside its hypomethylation and transcription in normal and tumor cells [72, 84, 171, 174]. Finally, a donor L1 may be repressed in most contexts (Fig. 3, yellow scenario) but, if located downstream of an active endogenous active promoter, transcription directed by this external promoter may initiate upstream of, and read through into, the L1, thereby generating an intact L1 mRNA. This arrangement could yield somatic L1 insertions with 5′ transductions [36, 69, 73] and may explain one of the examples described above in cortical neurons [135]. In principle, these scenarios present mechanistic bases for individual L1s escaping repression, being transcribed [84, 163, 195, 196], and producing somatic variants that are carried by mature differentiated cells where mobile L1 subfamilies are, overall, marked by epigenetic and transcriptional silencing [19, 20, 22, 27].

## Non-canonical L1-associated somatic genome variation

Despite proof of somatic retrotransposition in mammalian brain cells, L1 could impact neuronal phenotype via other routes. For example, a single-cell genomic analysis [134] of L1 insertions in the human hippocampus identified TPRT-mediated retrotransposition events, corroborating a previous study [137]. The authors also reported examples of somatic genome deletions flanked by germline L1 copies that were detectable in single cells but could also be PCR amplified in bulk hippocampus DNA via digital droplet PCR and PCR reactions performed on very high (500 ng) input template quantities [134]. These deletions were attributed to DNA damage associated with L1 endonuclease activity independent of retrotransposition [200]. Notably, the aforementioned WGS analysis of mouse olfactory neuron clones obtained by nuclear transfer [138] did not report L1-associated deletions, but also studied fewer neurons from a different species and neuroanatomical region. The frequency and distribution of L1-driven genomic deletion events in humans and other mammals therefore remain to be determined.

More recently, a WGS analysis of bulk human brain tissues [201] reported thousands of somatic L1 insertions although, surprisingly, the vast majority of these were found nested within L1 insertions annotated on the reference genome. This “L1-within-L1” scenario [202] presents a significant bioinformatic challenge as sequencing reads can align unreliably to highly repetitive regions [203], and for this reason insertions into existing younger L1 subfamily (e.g. L1-Ta, L1PA2) copies are usually filtered by TE insertion calling software [204]. Moreover, the putative somatic L1 insertions appeared to not involve L1 ORF2p endonuclease activity [44], and were 3′ truncated, a feature of L1 integration not encountered for canonical TPRT-mediated L1 insertions in normal cells, where 5′ truncation is instead common [205, 206]. The authors of this study verified a set of nested germline L1 insertions identified by their approach and a publicly available long-read sequencing dataset but, importantly, did not present a similar analysis of long-read sequencing applied to the same brain samples already analyzed by WGS, or sequence matched non-brain tissues [201]. Finally, the proprietary analysis tools required to identify TE insertions in sequencing data generated by this study, and other studies based on the Complete Genomics platform [26], significantly complicate data sharing and critical re-analysis. L1 may therefore alter the neuronal genome via unexpected pathways, but studies in this area require further investigation and replication, including additional validation and single-cell genomic analyses.

## Non-integrated L1 sequences in neural cells

Full-length L1 mRNA transcription can occur in the normal brain [19, 20, 195, 196]. As well as via DNA methylation, the L1 promoter is in this context regulated by a variety of transcription factors, including SOX2 (Fig. 1, Table 1) [20, 22, 27, 47, 69, 105, 207]. An antisense promoter is also present in the human L1 5′UTR [208], is conserved in primates, and has independent protein-coding potential [209]. This antisense promoter initiates transcription in numerous spatiotemporal contexts and can provide canonical promoters to protein-coding genes [117, 196, 208–212]. 5′ truncated L1s can also act as promoters in the brain, perhaps regulated by the Wnt signaling pathway [22, 196]. Thus, mobile and immobile L1 copies, where the latter are far more numerous, contribute various L1-initiated RNAs to the cellular environment. These can fulfill cis-regulatory roles and act globally to regulate chromatin structure [213, 214]. L1 transcription, protein abundance and mobilization rate may become uncoupled in vitro upon high L1 mRNA expression [215]. The production of diverse sense and antisense L1 RNAs, and their cellular abundance, may therefore in itself impact neuronal phenotype, independent of retrotransposition.

Similarly, L1 DNA sequences not integrated into the host genome, perhaps generated by ectopic reverse transcription primed from other cellular RNAs, aborted retrotransposition events, or another process involving the L1 machinery, may be relevant to cellular function [216–218]. Human and mouse L1 CNV assays applying multiplex qPCR to template DNAs extracted from tissue have repeatedly shown variation in L1 DNA content, when brain regions are compared to each other, and when brain samples are compared to non-brain tissue [20, 24, 25, 27, 133, 137, 219]. These studies suggest that i) the hippocampus is a hotspot for L1 CNV and ii) brain tissues are generally enriched for L1 DNA, versus non-brain tissues. As has been proposed previously [112, 220], qPCR-based L1 CNV assays cannot alone demonstrate retrotransposition because they do not discriminate L1 sequences that are, or are not, integrated into the genome. Host factor defenses against retrotransposition very likely include the degradation of single-stranded DNA intermediates produced during TPRT (Table 1) [112, 139] and, where this process is deficient, cells may accumulate single-stranded L1 DNA molecules [221]. Control experiments, such as enzymatically treating qPCR input templates to degrade single-stranded DNA, or selecting only high molecular weight DNA via gel electrophoresis, may reduce, but cannot exclude, the potential for non-integrated L1 DNA to dominate qPCR-based L1 CNV assays [25]. Indeed, these qPCR-based assays can also return absolute L1 CNV values reflecting hundreds of new L1 insertions per cell, depending on normalization approach, when all single-cell genomic analyses performed to date have shown retrotransposed products at a rate far lower than this [35, 67]. It is possible that the qPCR-based assays are simply confounded by unanticipated technical issues and are quantitatively unreliable. In our view, it is more plausible that, alongside L1 RNA expression, neurons can accumulate L1 DNA molecules that are not integrated into the nuclear genome.

The origin, composition and cellular impact of non-integrated L1 DNA sequences remain unclear. They may arise due to a failure to resolve or degrade TPRT intermediates, ectopic L1 reverse transcription where the products are sequestered in the cytosol, or another mechanism by which L1 could form stable, extrachromosomal DNA sequences in vivo [216–218, 221–227]. Are these L1 DNAs predominantly single- or double-stranded? Are they predominantly full-length or heavily truncated? Notably, qPCR assays targeting L1 at its 5′UTR, ORF2 or 3′UTR regions can in some cases generate different L1 CNV results [25, 27], suggesting that the additional L1 DNA sequences are shorter on average than genomic L1 copies of the same subfamily, which supports the hypothesis that interrupted, or unusually inefficient, reverse transcription may be involved in the biogenesis of non-integrated L1 DNA molecules. Along these lines, when the L1 qPCR assay was applied to brain tissue obtained from i) Rett syndrome (RTT) patients, where mutations in the L1 transcriptional repressor *MeCP2* (Table 1) [27, 75, 228, 229] cause a severe neurodevelopmental disorder, and ii) an *MeCP2*-mutant RTT mouse model, significant L1 copy number gain was observed in either species when L1 DNA content was measured at ORF2, when compared to controls [27]. L1 CNV was not, however, observed when measured at the 5′UTR [27]. It is relevant that conditional restoration of MeCP2 function in *MeCP2*-mutant mice leads to robust reversal of neurological phenotype [230]. In work performed recently in our laboratory, we found that phenotypic reversal in these animals was accompanied by L1 DNA content returning from elevated to wild-type levels after rescue, when measured by qPCR against ORF2 (Morell et al., *unpublished data*).

These observations altogether suggest that at least some of the additional L1 DNA content reported in RTT brain samples may not be incorporated into the nuclear genome. More broadly, the increased presence of L1 and other TEs in neurological disorders [6, 27–29, 231–234] elucidated by qPCR-based assays therefore may not involve new TE insertions, and any associated potential toxicity [235] may not be due to retrotransposition. It is tempting to speculate that the accumulation of non-integrated L1 DNA, for example via failed or incomplete elimination of TPRT intermediates [52, 139, 236], could still cause genomic lesions in neuronal genes [237] or otherwise “distract” host factors which, in addition to guarding against L1 integration, often regulate other cellular processes [112]. L1 activity in the brain is potentially relevant to neuronal physiology and genome stability beyond any impact of somatic retrotransposition, although further experiments are required to demonstrate the biogenesis of non-integrated L1 DNA sequences in neurons and other cells.

## Does elevated L1 content in the brain trigger autoimmunity?

Endogenous and exogenous nucleic acids may trigger immune responses mediated by various sensor pathways [for reviews, see [238, 239]]. As well as in RTT, elevated L1 DNA content has been reported in neurological disorders associated with autoimmunity, immunodeficiency and maternal infection, including Aicardi-Goutières syndrome [29, 137, 221], ataxia telangiectasia [74] and schizophrenia [26]. As for normal individuals, the magnitude of L1 CNV reported in these disorders appears to far exceed what would plausibly be due to somatic retrotransposition and could be due to an accumulation of L1 DNA molecules that are not integrated into the nuclear genome [240]. This scenario would have major implications for the treatment of any condition proven to be caused by L1 activity because the reversal of any associated symptoms would no longer be dependent on the challenging excision of somatic L1 insertions from neuronal genomes. Instead, processes leading to an accumulation of non-integrated single- or double-stranded L1 DNA could be targeted, for example, with reverse transcriptase inhibitors [241] or through targeted silencing [242] of heavily transcribed L1 copies [84].

Aicardi-Goutières syndrome (AGS) is a very rare interferonopathy that provides arguably the best developed example of a neurological phenotype potentially linked to L1-associated autoimmunity. Genetic analyses of AGS patients have revealed mutations most commonly in the genes *TREX1*, *SAMHD1*, *ADAR1, RNASEH2A*, *RNASEH2B*, *RNASEH2C* and *IFIH1* [239, 243]. Most of these genes encode factors that have been shown to regulate retrotransposon activity (Table 1) [221, 234, 244–251], supporting the hypothesis that the cytosolic accumulation of endogenous nucleic acids in AGS generates an interferon response [239, 252–254]. TREX1, for example, is an established exonuclease of aberrant single-stranded intermediates generated during DNA replication [255]. An abundance of single-stranded L1 DNA has been reported in human and mouse TREX1-deficient cells [29, 221], whilst a single-cell genomic analysis of neurons obtained from one AGS patient carrying *SAMHD1* mutations indicated that somatic L1 insertions occurred at a rate similar to that of controls [137]. Whilst these experiments suggest L1 might play a role in AGS, the mechanism via which single-stranded L1 DNA could generate an abnormal neuronal phenotype is largely unclear, and it remains plausible that the accumulation of L1 DNA in AGS is a largely inconsequential result of nuclease mutations.

Intriguingly, a recent study demonstrated that media obtained from TREX1-deficient human astrocytes was toxic to healthy neurons, whereas media from TREX1-deficient astrocytes treated with L1 reverse transcriptase inhibitors was significantly less toxic [29]. The authors ascribed this toxicity to an interferon response due to an accumulation of cytosolic single-stranded L1 DNA in astrocytes [29, 256]. By contrast, another recent work found that treatment of *TREX1* mutant mice with L1 reverse transcriptase inhibitors had no impact on interferon response or the retrotransposition frequency of an engineered L1 reporter gene in vivo [257]. Previously, different reverse transcriptase inhibitors have been shown to rescue [258] or not rescue [221] the lethal myocarditis phenotype of TREX1-deficient mice. These findings raise the prospect that a biochemical mechanism apart from the inhibition of L1 reverse transcriptase activity, perhaps instead targeting inflammation, is responsible for the amelioration of AGS phenotype [259].

At this stage, the etiological role of *TREX1* in controlling L1 and other endogenous retrotransposons in AGS requires further study. It should however be noted that i) the somewhat opposing results detailed above for L1 were obtained using different species and cell types, ii) assays measuring engineered and endogenous L1 activity can provide different results [29, 221, 247, 257], iii) engineered L1 retrotransposition frequency and potentially immunogenic single-stranded L1 DNA content are not equivalent, and iv) host factors and reverse transcriptase inhibitors may act via multiple direct and indirect pathways to limit L1 activity. For example, instead of restricting L1 primarily by exonuclease activity, TREX1 may alter the subcellular localization of L1 ORF1p, and thereby reduce opportunities for cells to accumulate L1 DNA, whether via retrotransposition or another mechanism [221, 247].

As for TREX1, RNaseH2 has been alternatively reported as being a negative or positive regulator of L1 retrotransposition [249, 250, 260]. Some eukaryotic TEs encode ribonuclease proteins to facilitate the removal of their template RNA after reverse transcription [261–263], and also degrade other cellular DNA:RNA hybrids, supporting a positive role for RNaseH2 in L1 retrotransposition. Alternatively, biochemical assays using the *Bombyx mori* R2 retrotransposon previously revealed that the RNA in a hybrid DNA:RNA molecule generated during TPRT could be displaced during second strand DNA synthesis without the apparent involvement of a ribonuclease [264]. Ribonuclease mediated degradation of the RNA strand of hybrid L1 DNA:RNA molecules prior to second strand synthesis has been demonstrated in vitro to expose the L1 cDNA to deamination, suggesting that ribonuclease activity may facilitate editing or 5′ truncation of L1 cDNAs in vivo [139]. Nonetheless, we favor the view that the ribonuclease activity of RNaseH2 assists L1 mobility in vivo, even if other RNaseH2 functions are ultimately shown to inhibit retrotransposition. Overall, the available literature points to a potential role for L1 in the etiology and clinical management of AGS and other neurodevelopmental disorders associated with autoimmunity. Significant work is required to reconcile the somewhat opposing results reported for the use of reverse transcriptase inhibitors in disparate AGS experimental models, and to therefore clarify whether L1 activity is a pathogenic or coincidental feature of this disease.

## Conclusions

Somatic mosaicism represents an intriguing and underexplored form of genetic and biological variation in mammals. Although L1 retrotransposon-driven mosaicism is now established to occur in brain cells, any impact of this phenomenon upon normal and abnormal neurobiological processes remains undemonstrated. Despite the recent development of tools, including single-cell genome, epigenome and transcriptome sequencing [151, 265–272], in some cases employed in parallel [for a review, see [273]], as well as CRISPR-Cas9 based genetic and epigenetic engineering [242, 274–277], conclusive proof is yet to be provided of any individual somatic L1 insertion arising in the neuronal lineage that has generated a molecular, biochemical or behavioral phenotype in vivo. Given the effect size of L1 insertions in genes, and the frequency of endogenous L1 insertions arising during neurodevelopment, adult neurogenesis or in post-mitotic neurons, it is likely that some L1 insertions could induce a biologically relevant neuronal phenotype. We believe such examples will be found in future studies. It is also plausible that L1 may impact neurobiology primarily through mechanisms not involving resolved retrotransposition events, given recent observations from neurological diseases, such as RTT and AGS.

Experiments to test the impact of individual somatic L1 insertions present a major challenge. Work in this area could be greatly accelerated through: i) the development of methods to reliably survey genome structural variation and transcription, genome-wide and from the same cell, using human brain tissue obtained post-mortem, or from tissue obtained during brain surgery [278, 279], or from animal models, ii) the large-scale production of WGS data from individual brain cells, retaining neuronal subtype information, as well as from non-brain cells, and iii) the ability to introduce, via CRISPR-Cas9 or another approach, L1 insertions found in vivo into cultured neurons, organoids or even animal models, to assess their impact upon the transcriptional and regulatory landscapes when established in a homogenous cellular population. Long-read sequencing approaches, such as those developed by PacBio and Oxford Nanopore, which can identify TPRT hallmarks ab initio by resolving L1 integration sites in full, may also prove particularly useful, even if simply applied at high depth to DNA extracted from brain tissue [280–284]. Beyond surveying the spatiotemporal extent and potential immediate functional impact of L1 mosaicism, we also need to be able to modulate endogenous retrotransposition and evaluate the consequences, if any, upon behavior. In neurological disorders where elevated L1 activity is apparent, it would be valuable to assess the impact restricting that activity has upon symptoms. These are long term and challenging experiments. However, neuronal genome mosaicism driven by engineered L1 retrotransposition was first reported in 2005 [21] and has only been definitively shown to be recapitulated by endogenous L1s in vivo quite recently [133–138]. Therefore, equipped with foundational knowledge, and improving tools, the field is well positioned to move rapidly towards establishing any functional impact of L1 mosaicism in the soma.

## Abbreviations

AGS: Aicardi-Goutières syndrome
CNV: Copy number variation
CRISPR: Clustered regularly interspaced short palindromic repeats
EGFP: Enhanced green fluorescent protein
LINE-1 (or L1): Long interspersed element-1
ORF: Open reading frame
polyA: Polyadenine
qPCR: Quantitative PCR
RTT: Rett syndrome
SNV: Single nucleotide variant
TE: Transposable element
TPRT: Target-primed reverse transcription
TSD: Target site duplication
UTR: Untranslated region
WGA: Whole-genome amplification
WGS: Whole-genome sequencing
